# Readmission Rate After Methicillin-Resistant Staphylococcus aureus (MRSA) Infection: A Retrospective Study

**DOI:** 10.7759/cureus.80280

**Published:** 2025-03-09

**Authors:** Riddhi Patel, Sun Moon Kim, Yarl Dhayaparan, Leila Bouchekouk, James Letsen, Khin Oo, Bhawaninder Kaur, Monica Lee, Mark Samarneh

**Affiliations:** 1 Internal Medicine, Lake Erie College of Osteopathic Medicine, Yonkers, USA; 2 Internal Medicine, St. John's Riverside Hospital, Yonkers, USA; 3 Internal Medicine/Nephrology, Riverside Health System, Yonkers, USA

**Keywords:** 30-day readmission, colonization, comorbidity control, healthcare burden, hospital readmission rate, infectious disease control, methicillin resistant staphylococcus aureus (mrsa)

## Abstract

Background

The exponential increase in the incidence of Methicillin-resistant* Staphylococcus aureus *(MRSA) infections has imposed great burdens on hospital systems. Although there are vast antibiotic options for the treatment of MRSA, eradication is often tough. There are not many studies highlighting the post-discharge readmission rates for MRSA patients.

Objective

The primary aim of the study was to describe the 30-day readmission rate for patients hospitalized with a positive MRSA culture from a single center, with a secondary aim of analyzing 90-day readmission rates.

Methods

The authors conducted a retrospective study based on a chart review of patients’ electronic medical records and the 3M billing platform to obtain information regarding patients treated for MRSA infections. Inclusion criteria were age above 18, admission to a single center in the United States, and positive microbial MRSA diagnoses between 2014 and 2024. Data regarding demographics, comorbidities, length of stay, and readmission diagnoses were collected and analyzed using Microsoft Excel Version 16.91.

Results

The retrospective analysis revealed a total of 127 patients who met the inclusion criteria. The demographics of the patient population were predominantly male and over the age of 65. Among the patients, 17.32% (22 out of 127 patients) had a readmission within 30 days of discharge after a MRSA infection. Of these 22 patients, 50% were male and 50% were female, with an average age of 65. These 22 patients accounted for 24 readmissions within 30 days of discharge. Of all the readmissions, 54.17% (13/24) were due to an infectious process, and 45.83% (11/24) were due to the exacerbation of comorbidities. Only 1 readmission was a result of recurrent MRSA bacteremia. The average readmission ratio within 30 days of MRSA infection was 1.09 per patient.

Among the 127 patients included in the study, 27.56% (35/127) were readmitted at least once to the hospital within 90 days following discharge for the treatment of initial presentation with a positive MRSA culture. However, these 35 patients accounted for a total of 54 readmissions within 90 days of discharge. Of all the readmissions, 44.44% (24/54) were due to an infectious cause, while 44.44% (24/54) were due to the exacerbation of comorbidities. The average readmission ratio within 90 days of MRSA infection was 1.5 per patient.

Conclusion

The high readmission rates for patients with MRSA can be attributed to the increased morbidity associated with MRSA. Approximately half of the patients who were readmitted within 30 days were admitted due to an infectious process, whereas the other half were admitted due to the exacerbation of comorbidities.

## Introduction

Infections due to *Staphylococcus aureus *have been proven to be increasingly prevalent throughout the United States. The incidence and prevalence of Methicillin-resistant *Staphylococcus aureus* (MRSA) infections skyrocketed in the 90s and have only continued to grow [1]. MRSA infections are notoriously hard to eradicate and result in increased healthcare costs and burdens not only for the patient but also for the hospitals. 

Patients with MRSA infections have significantly longer hospital stays relative to unafflicted patients, contributing to increased healthcare resource utilization [1-8]. Additionally, it has been found that patients who contracted MRSA at a surgical site increased their hospital length of stay by a mean of 5 additional days. MRSA infections result in larger medical expenses due to the longer length of stay and increased resource utilization. Perhaps of more concern, Engemann et al. also found an increased mortality rate for surgical patients who contracted MRSA surgical site infections [5].

In addition to the financial burden, MRSA infections have increased transmission risk, higher antibiotic resistance rate, increased surgical complications, and increased infection rate in inpatient and outpatient settings [9]. Researchers have shown that around half of the population infected with MRSA had a median clearance time of 7.4 months [10].

Across the nation, the 30-day readmission rate is 22% for MRSA bacteremia, and the recurrence of bacteremia is higher in patients with MRSA than Methicillin-sensitive Staphylococcus aureus (MSSA). MRSA bacteremia is also associated with increased morbidity and mortality when compared to MSSA and is commonly found in patients with endocarditis, immunocompromised states, and intravenous drug use. Not only did comorbidities such as chronic obstructive pulmonary disease (COPD), congestive heart failure (CHF), and human immunodeficiency virus (HIV) impact readmission rates, but socioeconomic factors also had an impact [1]. Patients who resided in a low-income neighborhood, who had Medicare or Medicaid insurance, and who were treated in a non-teaching hospital had poorer outcomes and an increased rate of readmission [1]. Another study revealed that the 90-day readmission rate was approximately 46% for patients with MRSA infection [11]. These patients were admitted to the medicine team, surgical team, or ICU. Additionally, readmission of asymptomatic MRSA carriers in a study by Inagaki et al. contributed to the high readmission rates seen and thus also contributes to the large burdens placed on healthcare systems [1].

The aim of this study was to provide an estimate of the readmission rate seen in a single center in the United States and to understand the causality behind readmissions post MRSA infection. Although readmissions rates are high following MRSA infections, there are not many studies highlighting the post-discharge readmission rates for MRSA patients. By understanding the underlying causes of readmissions, we can potentially prevent many of the readmissions by optimizing management within the hospitalization, discharge planning, and post-discharge management.

## Materials and methods

Study design

This study was a retrospective study based on a chart review from St. John’s Riverside Hospital system medical records of patients who are admitted to the hospital with positive MRSA colonization regardless of diagnosis and readmitted to the hospital within 30 and 90 days of discharge. The study was conducted by accessing the electronic medical records (EMR) and 3M billing platform to create a report of patients diagnosed with MRSA infection. The report did not contain any patient identifiers except for the patient's EMR number to allow access to the profile. The report was stored on a password-protected database and entered on a computer only accessible from St. John's Riverside Hospital. The report included all patients aged 18 years and older with a positive MRSA diagnosis between January 1st, 2014, and August 31st, 2024. MRSA will be defined based on the Clinical Laboratory Standards Institute (CLSI) standard as an oxacillin minimum inhibitory concentration (MIC)≥4 mcg/mL [12]. All patients in the report were subjected to a systemized screening process to exclude patients without a positive MRSA culture in the EMR's microbiology tag. The inclusion criteria were patients who were above the age of 18 and who had a confirmed MRSA infection. Exclusion criteria were an age less than 18. The electronic charts of subjects who met the inclusion and exclusion criteria were accessed using St. John’s Riverside in-patient registry. Demographics (gender, age, race), comorbidities, history of MRSA infection, inpatient treatment diagnoses, total length of stay, disposition, readmission frequency, and readmission diagnoses were recorded. Since this study is retrospective, there is no risk to patients. Thus, a consent waiver form was not required. The proposal underwent a thorough review by a St. John's Riverside institutional review board to ensure the highest ethical standards were met.

Statistical testing

The authors conducted statistical analyses using Microsoft Excel Version 16.91 (24111020). Chi-square tests and t-tests were used to assess the data, and a confidence level of 95% was used for all analyses. The primary points of interest were the 30-day readmission rates following discharge from MRSA infection and the underlying etiology of readmission, which encompassed infection, exacerbation of comorbidities, and other causes. Secondary outcomes included the 90-day readmission rates and further quantification of the impact of comorbidities. 

## Results

Between 2014 and 2024, we identified 127 patients who met the inclusion criteria of having a positive MRSA culture. The patients were predominantly male with an average age of 68.7±18.6 years. 22 (17.32%) patients were readmitted within 30 days of discharge, and of those patients, two patients were readmitted multiple times (9.09%, p=1) (Table 1). The average age of the patients readmitted within 30 days was 65 (p=0.468, 95% CI [-11.08 to 23.26], SE 8.232). Within 30 days of discharge after a MRSA infection, 17.32% (p=0.261) experienced readmissions, contributing to 24 total readmissions. Of these, 54.17% were due to infectious causes, and 45.83% were due to the exacerbation of comorbidities (p=0.431), with only one case of recurrent MRSA bacteremia. The average readmission ratio within 30 days of MRSA infection was 1.09 per patient. Of the patient population that was readmitted within 30 days, further characterization revealed that there was no significant difference in the type of comorbidities exacerbation the patient presented with.

**Table 1:** Characteristics of 30 day readmissions. p-values were obtained using chi-square test, *significant at p<0.05.

Characteristics	Male, N (%)	Female, N (%)	p-value	X^2 ^-value
Readmitted	11 (8.66%)	11 (8.66%)	0.261	1.2600
Not readmitted	66 (51.97%)	39 (30.71%)		
Readmitted once	10 (45.45%)	10 (45.45%)	1	0
Readmitted multiple times	1 (4.54%)	1 (4.54%)		
Readmitted cause				
Infectious	5 (20.83%)	8 (33.33%)	0.431	0.6210
Exacerbation of comorbidities	6 (25.00%)	5 (20.83%)		
Comorbidity type				
Cardiovascular	0 (0.00%)	1 (9.09%)	0.517	1.32
Pulmonary	3 (27.27%)	2 (18.18%)		
Other	2 (18.18%)	3 (27.27%)		

Of the 22 (17.32%) patients who were readmitted, 13.6% were under the age of 40, 22.7% were between the ages of 40 and 65, and 63.6% of patients were above the age of 65. There was no significant difference in the readmission rate between age groups (p=0.760) (Table 2). Of the 24 total readmissions, 4.17%, 20.83%, and 29.17% of cases were due to readmission as a result of an infectious cause in the age groups of<40, 40-65, and >65 respectively, and 8.33%, 4.17%, and 33.33% were due to exacerbations of comorbidities in the age groups of <40, 40-65, and 65% respectively. However, readmissions as a result of any cause in any age group were not significantly different (p=0.232). Of the patients who were readmitted within 30 days, there was no significant difference in the types of comorbidities the patients had (p=0.868).

**Table 2:** Characteristics of 30 day readmissions based on age. p-values were obtained using a chi-square test, *significant at p<0.05.

Characteristics	≤40, N (%)	40-65, N (%)	≥65, N (%)	p-value	X^2^-value
Readmitted	3 (2.36%)	5 (3.94%)	14 (11.02%)	0.760	0.5499
Not readmitted	9 (7.09%)	26 (20.47%)	70 (55.12%)		
Readmitted once	2 (9.09%)	5 (22.73%)	13 (59.09%)	0.260	2.6976
Readmitted multiple times	1 (4.54%)	0 (4.17%)	1 (4.54%)		
Readmission cause					
Infectious	1 (4.17%)	5 (20.83%)	7 (29.17%)	0.232	2.9203
Exacerbation of comorbidities	2 (8.33%)	1 (4.17%)	8 (33.33%)		
Comorbidity type					
Cardiovascular	0 (0.00%)	0 (0.00%)	1 (9.09%)	0.868	1.2604
Pulmonary	0 (0.00%)	1 (9.09%)	3 (27.27%)		
Other	1 (9.09%)	1 (9.09%)	4 (36.36%)		

Among the 127 patients included in the study, 27.56% (35/127, p=0.894) were readmitted at least once to the hospital within 90 days following discharge for the treatment of initial presentation with a positive MRSA culture. The average age of the patients readmitted within 90 days was 66 (p=0.2792, 95% CI [-6.01 to 20.16, SE 6.430]) (Table 3). Of these 35 patients, 12 patients were admitted multiple times (p=0.042). However, these 35 patients accounted for a total of 54 readmissions within 90 days of discharge. Of all the readmissions, 46.30% (25/54) were due to an infectious cause, while 44.44% (24/54) were due to the exacerbation of comorbidities. The average readmission ratio within 90 days of MRSA infection was 1.5 per patient. 

**Table 3:** Characteristics of 90 day readmissions. p-values were obtained using chi-square test, *significant at p<0.05.

Characteristics	Male, N (%)	Female, N (%)	p-value	X^2^-value
Readmitted	21 (16.54%)	14 (11.02%)	0.894	0.0178
Not readmitted	54 (42.52%)	38 (29.92%)		
Readmitted once	11 (31.43%)	12 (34.29%)	0.042^* ^​​	4.1425
Readmitted multiple times	10 (28.57%)	2 (5.71%)		

## Discussion

The study underscores the significant burden of readmissions among patients hospitalized with MRSA infections. Although this study only focused on a single center in the United States, the alarmingly high 30-day readmission rate of 17% emphasizes the urgent need for enhanced management and prevention strategies for MRSA infections. Additionally, this study highlights the necessity for targeted post-discharge care initiatives for this vulnerable population. Notably, while the primary objective of this specific study was to assess the 30-day readmission rates, the 90-day readmission rates were also high at 27.56%. 

Demographic data shows that the average patient age is 68 years, with 59% of them being male. This older demographic likely contributes to a heightened burden of comorbidities, which healthcare providers must consider when developing management plans. Within the 30-days of discharge, approximately half of the patients (45.83%) experienced exacerbation of comorbidities leading to readmission. 

Lukovac et al. have noted that various comorbidities, such as cardiovascular diseases, malignancies, HIV, and diabetes, can lead to increased rates of MRSA infections and subsequent readmissions due to the patients’ immunocompromised states [13]. Comorbidities such as coronary artery disease, prior histories of myocardial infarction, cerebrovascular accidents, hypertension, diabetes, and chronic obstructive pulmonary disease were evident among the subject population. Further research must be conducted to understand how the different comorbidity impacts readmissions in this population [13].

While effective initial management of MRSA infections is critical, long-term management of associated chronic conditions is equally important for minimizing readmission rates. As seen in the study, approximately 50% of the patients experienced a readmission as a result of an exacerbation of a comorbidity. Ensuring that these comorbidities are effectively managed can reduce further hospital visits and subsequent readmissions. This dual focus can inform the development of comprehensive care plans that address not just the infection but also the patient's overall health. Implementing rigorous follow-up protocols and infection control measures within the first month after discharge may help mitigate these risks.

The Centers for Medicare and Medicaid Services (CMS) reported that in 2018, there were 2.3 million readmissions within 30 days in the United States, leading to costs totaling $35.7 billion [14]. Furthermore, approximately 1 in 10 of these readmissions were possibly preventable [14]. This notion can be expanded to the readmission rates seen in the study, as a substantial portion of readmission were due to exacerbations of comorbidities. To effectively reduce readmissions, it is crucial to enhance management practices, particularly in discharge planning, as well as to address the underlying factors contributing to preventable readmissions. By optimizing discharge planning and ensuring factors that contribute to preventable admission are addressed, readmission rates after a MRSA infection could be reduced; however, further research must be conducted. 

Social determinants of health play a significant role in influencing healthcare management and delivery. Generally, patients from lower socioeconomic backgrounds, underserved communities, or ethnic or racial minority groups tend to experience higher rates of 30-day readmissions. Five key categories of social determinants must be considered: economic stability, access to and quality of education, access to and quality of healthcare, environmental factors, and social/community relationships [14]. Additionally, the presence of comorbidities increases the likelihood of readmissions, underscoring the necessity for effective post-discharge follow-up and management of comorbidities. 

The higher readmission rates cannot be attributed solely to social determinants of health; rather, they are also influenced by factors such as inadequate discharge planning and ineffective patient communication during transition of care. When preparing for discharge, physicians should be cognizant of barriers to healthcare that patients may encounter, such as healthcare deserts (geographic locations where access to healthcare is limited) and challenges in obtaining medications. Equally important is ensuring that patients fully understand their discharge instructions [14].

The presence of healthcare deserts and limited access to primary care physicians greatly increases the risk of readmissions, as the lack of access to medical care impedes adequate patient follow-up post-discharge and negatively impacts patients’ health in the long term. Without shared decision-making and patient comprehension regarding the discharge plan, the unique needs of the patient will not be addressed, which will further contribute to higher readmission rates. 

Thus, physicians must engage in conversations with their patients to understand their preferences regarding care, while also considering patients’ cultural beliefs and values. A patient’s care plan must be tailored to incorporate these dimensions, fostering greater patient satisfaction and adherence and ultimately reducing the likelihood of readmissions.

One effective strategy for addressing the elevated rates of readmission is the implementation of a health-related social needs (HRSNs) screening tool. HRSNs encompass adverse social conditions that can negatively impact a patient’s health and access to care. Examples of HRSNs include food insecurity, housing instability, unemployment, limited access to affordable healthcare, inadequate education, and barriers to transportation [15].

Utilization of the HRSN screening tool enables physicians to tailor their care and recommendations to better align with each patient’s unique needs, thereby enhancing adherence to management plans. Moreover, it allows physicians to identify obstacles that may hinder effective healthcare delivery, facilitating discharge planning in collaboration with an interdisciplinary team. This screening tool should be implemented either upon admission or throughout the hospital stay to ensure that discharge plans incorporate social determinants of health and health-related social needs. 

Future research encompassing multiple centers and diverse demographics is essential for validating these results and understanding the variations in readmission patterns. Given the small amount of data available for analysis and the limitation of the billing software and EMR to generate a list of patients, further research with a larger sample size is necessary to determine the significance of the results. Furthermore, given the large presence of comorbidities, studies need to explore the effects of various comorbidities on the post-MRSA infection readmission rates. Additionally, future studies should prioritize developing and implementing interventions that address the management of MRSA infections and the holistic care of comorbid conditions, aiming to reduce overall readmission rates and improve patient outcomes. 

## Conclusions

MRSA infections pose large burdens, medically and financially, on the patient and the healthcare system. The high antibiotic resistance of the bacterial infection results in a longer duration of treatment, poorer outcomes, and higher financial costs for the patients. Additionally, patients with comorbidities have a poorer prognosis but also have a higher risk of readmission due to their tumultuous states.

The authors saw that approximately half of the patients who were readmitted were readmitted due to the exacerbation of comorbidities. Strategies to address negative socioeconomic factors that impact overall health must be used during inpatient stays and upon discharge to ensure that the risk of readmission is minimized. Additionally, management of comorbidities must be done holistically and in conjunction with the interdisciplinary team to ensure all aspects of the patients' health and limiting factors are addressed to reduce the risk of readmission.

## References

[1] Inagaki K, Lucar J, Blackshear C, Hobbs CV (2019). Methicillin-susceptible and methicillin-resistant Staphylococcus aureus bacteremia: Nationwide estimates of 30-day readmission, in-hospital mortality, length of stay, and cost in the United States. Clin Infect Dis.

[2] Tissot F, Calandra T, Prod'hom G, Taffe P, Zanetti G, Greub G, Senn L (2014). Mandatory infectious diseases consultation for MRSA bacteremia is associated with reduced mortality. J Infect.

[3] Allareddy V, Das A, Lee MK, Nalliah RP, Rampa S, Allareddy V, Rotta AT (2015). Prevalence, predictors, and outcomes of methicillin-resistant Staphylococcus aureus infections in patients undergoing major surgical procedures in the United States: a population-based study. Am J Surg.

[4] Patel M, Weinheimer JD, Waites KB, Baddley JW (2008). Active surveillance to determine the impact of methicillin-resistant Staphylococcus aureus colonization on patients in intensive care units of a Veterans Affairs Medical Center. Infect Control Hosp Epidemiol.

[5] Engemann JJ, Carmeli Y, Cosgrove SE (2003). Adverse clinical and economic outcomes attributable to methicillin resistance among patients with Staphylococcus aureus surgical site infection. Clin Infect Dis.

[6] Hanberger H, Walther S, Leone M (2011). Increased mortality associated with methicillin-resistant Staphylococcus aureus (MRSA) infection in the intensive care unit: results from the EPIC II study. Int J Antimicrob Agents.

[7] Huang SS, Singh R, McKinnell JA (2019). Decolonization to reduce postdischarge infection risk among MRSA carriers. N Engl J Med.

[8] Self WH, Wunderink RG, Williams DJ (2016). Staphylococcus aureus community-acquired pneumonia: prevalence, clinical characteristics, and outcomes. Clin Infect Dis.

[9] Raygada JL, Levine DP (2009). Methicillin-resistant Staphylococcus aureus: a growing rsk in the hospital and in the community. Am Health Drug Benefits.

[10] Marschall J, Mühlemann K (2006). Duration of methicillin-resistant Staphylococcus aureus carriage, according to risk factors for acquisition. Infect Control Hosp Epidemiol.

[11] Grohs P, Pineau B, Kac G, Gutmann L, Meyer G (2013). Readmission of known MRSA carriers and MRSA colonization pressure in hospital. Epidemiol Infect.

[12] Siddiqui AH, Koirala J (2025). Methicillin-Resistant Staphylococcus aureus [Updated 2023 Apr 2]. https://www.ncbi.nlm.nih.gov/books/NBK482221/.

[13] Lukovac E, Koluder-Cimic N, Hadzovic-Cengic M, Baljic R, Hadzic A, Gojak R (2012). Analysis of comorbidity of the patients affected by staphylococcal bacteremia/sepsis in the last ten years. Mater Sociomed.

[14] (2024). CMS office of minority health. Guide for reducing disparities in readmissions. Centers for Medicare and Medicaid Services. https://www.cms.gov/about-cms/agency-information/omh/downloads/omh_readmissions_guide.pdf.

[15] (2024). Accountable health communities. A guide to using the accountable health communities health-related social needs screening tool: Promising practices and key insights. Centers for Medicare and Medicaid Services. https://www.cms.gov/priorities/innovation/media/document/ahcm-screeningtool-companion.

